# Lessons Learned in Protein Precipitation Using a Membrane Emulsification Technique to Produce Reversible and Uniform Microbeads

**DOI:** 10.3390/pharmaceutics13101738

**Published:** 2021-10-19

**Authors:** Sang-Koo Park, Ga Yeon Noh, Hyun Woo Yu, Eun Chae Lee, Junoh Jeong, Young-Min Park, Hyo-Kyung Han, Seong Hoon Jeong, Nam Ah Kim

**Affiliations:** 1 BK21 FOUR Team and Integrated Research Institute for Drug Development, College of Pharmacy, Dongguk University, Seoul 10326, Korea; skpark1973@gmail.com (S.-K.P.); shrkdus96@naver.com (G.Y.N.); yhw903@naver.com (H.W.Y.); eun_chae527@naver.com (E.C.L.); junoh0621@naver.com (J.J.); hkhan@dongguk.edu (H.-K.H.); 2 Division of Health and Kinesiology, Incheon National University, Incheon 22012, Korea; ypark@inu.ac.kr

**Keywords:** membrane emulsification, protein stability, protein aggregation, SPG membrane, trehalose, intravenous IgG (IVIG), microbead

## Abstract

The effects of the manufacturing process and the regeneration of Shirasu porous glass (SPG) membranes were investigated on the reproducibility of protein precipitants, termed protein microbeads. Intravenous immunoglobulin (IVIG) was selected as a model protein to produce its microbeads in seven different cases. The results showed that the hydrophobically modified SPG membrane produced finer microbeads than the hydrophilic SPG membrane, but this was inconsistent when using the general regeneration method. Its reproducibility was determined to be mostly dependent on rinsing the SPG membrane prior to the modification and on the protein concentration used for emulsification. The higher concentration could foul and plug the membrane during protein release and thus the membrane must be washed thoroughly before hydrophobic modification. Moreover, the membrane regenerated by silicone resin dissolved in ethanol had better reproducibility than silicone resin dissolved in water. On the other hand, rinsing the protein precipitant with cold ethanol after the emulsification was not favorable and induced protein aggregation. With the addition of trehalose, the purity of the IVIG microbeads was almost the same as before microbeadification. Therefore, the regeneration method, protein concentration, and its stabilizer are key to the success of protein emulsification and precipitation using the SPG membrane.

## 1. Introduction

Protein precipitation is gaining interest for downstream steps in bioprocesses due to its capability of purifying therapeutic proteins, including monoclonal antibodies (mAbs) and immunoglobulins (IgGs) in a scalable and cost-effective manner [1,2,3,4]. Implementational studies of bioprocesses have been increasing, with more publications as well as an influx of patent submissions [1,5]. The bioprocess covers changing proteins from a liquid to a solid state by decreasing their solubility, which can be induced by various agents, e.g., neutral salts, organic solvents, nonionic polymers, polyelectrolytes, acids, and affinity ligands [1]. However, the protein stability during precipitation should be carefully considered since dehydration of the protein may lead to protein unfolding, and non-native or irreversible protein aggregation may also occur. Moreover, the aggregates or proteinaceous particles are known to cause adverse immunogenicity [6,7,8,9,10,11,12].

Organic solvent-based protein precipitation for sample preparation prior to mass spectrometry has also been widely used in proteomic analysis for almost a century, eliminating interferences with high protein recovery [13,14,15]. An increasing salt concentration and incubation temperature in 80% acetone with rapid precipitation have resulted in high protein recovery (around 98%) from complex proteome extracts [16]. Based on these findings, we hypothesized that organic solvents are promising for producing reversible protein precipitates, but they require new insights into their mechanisms for designing better preparation methods that are applicable for commercial production. In our previous study, a combination process of cold *n*-octanol precipitation with membrane emulsification was able to produce a uniform and reversible IgG precipitant (called a ‘microbead’) [17]. Briefly, IgG solution was injected into cold *n*-octanol through a Shirasu porous glass (SPG) membrane to produce a uniformly distributed water-in-oil (W/O) emulsion followed by vortexing for rapid precipitation (i.e., dehydration). Then, it was centrifuged to remove the supernatant and vacuum dried under controlled vapor pressure to remove any remaining organic solvents. With this effort to improve the process development, the recovery of IgG upon rehydration exhibited almost the same content as before the precipitation process [17].

SPG membrane is often used for small molecules for the preparation of emulsions, microspheres, microcapsules, and microparticles [18]. It is inherently hydrophilic because of the presence of silanol groups on its surface, which is not suitable for W/O emulsification. Treatment with silicone resin (i.e., KP-18C; a C_18_ hydrophobic chain) can change the surface of the membrane by reacting with the Si-OH group of the membrane to become more hydrophobic. Regeneration of the membrane has been reported to allow it to be repeatedly used with almost the same emulsification performance as freshly modified membranes [19]. The first study on utilizing membrane emulsification technique can be traced back to the later 1980s [20,21]. However, there are only a limited number of studies on its applications for biologicals and they were conducted mostly on bovine serum albumins (BSA), which is relatively more stable than the other therapeutic proteins [18,22,23,24].

Herein, we report the impact of hydrophilic and regenerated hydrophobic SPG membrane on its reproducibility and the reversibility of the protein precipitant (i.e., microbeads) after a protein emulsification-precipitation process (Table 1). For the model protein, marketized intravenous IgG (IVIG), somewhat prone to aggregate by interfacial stresses [25], was evaluated with different pore sizes and lot numbers of membranes. Moreover, the impact of two different solvent-based silicone resin solutions (i.e., deionized water- and ethanol-based KP-18C solution) for regeneration of the SPG membrane was also investigated. In summary, the seven different case studies listed in Table 1 were comprehensively interpreted in this study.

## 2. Materials and Methods

### 2.1. Materials and Dialysis

The 10% intravenous immunoglobulin (IVIG) consisting of 18.8 mg glycine and 100 mg IgG per 1 mL (Trade name: IV-GlobulinSN Inj. 10%; Lot number: 383A19001) was purchased from Green Cross (Gyeonggi, Korea) and the same lot was used throughout the study. Sodium acetate trihydrate, acetic acid, and glycine were purchased from Sigma-Aldrich (St. Louis, MO, USA) to prepare 10 mM sodium acetate buffer at pH 4.0 with and without glycine for dialysis medium. IVIG was loaded in a Slide-A-Lyser^®^ Dialysis Cassettes with a 10 kDa molecular weight cut-off (Thermo Scientific, Rockford, IL, USA), and it was inserted in one liter of dialysis buffer and stirred at 50 rpm in a refrigerator. The dialysis buffer was replaced twice at eight-hour intervals. Biological grade polysorbate 80 (PS80) was also from Sigma-Aldrich (St. Louis, MO, USA), and trehalose was supplied from Pfanstiehl Inc. (Waukegan, IL, USA). The additives were dissolved in the same buffer and spiked into the dialyzed samples. Prior to the determination of its concentration, the prepared solutions were filtered using a sterile cellulose acetate centrifuge tube filter (Spin-X 0.22 µm, Costar, Corning Inc., Salt Lake, UT, USA) at 8000 rpm for two min. *n*-Octanol was obtained from Junsei Chemicals (Tokyo, Japan) and all other reagents used were of analytical grades.

### 2.2. IVIG Microbead Preparation Using the SPG Membrane

An internal pressure type micro kit (IMK-20; MCtech, Siheung, Korea) was adopted as a dispersion-emulsifying system. It was utilized to emulsify the IVIG solution in cold *n*-octanol stored at around 2–8 °C. Briefly, different pore-size (i.e., 1.5 μm, 3 μm, and 5 μm) and 20 mm (w) × 10 mm (h)) of tube-shaped SPG membrane (SPG Techno Co., Ltd., Miyajaki, Japan) was clamped with O-rings (AN-008; MCtech, Siheung, Korea) and screwed into the membrane module. Prior to emulsification, IVIG solution was filled in the dispersed phase and cold *n*-octanol as the continuous phase outside the SPG membrane. N_2_ gas was purged at a pressure of 50 kPa to release the IVIG solution. Similar to the previous study [17], the volume ratio of IVIG solution and *n*-octanol was derived from a water saturation fraction (*f*) which was fixed at 0.5 for fast dehydration [26,27,28]. The volume of the IVIG solution, *V_w_*, was calculated according to the following equation:

*V_w_* = *f* × *V_s_* × *C_s_* × *ρ*

where *V_s_* is the volume of *n*-octanol, *C_s_* is the solubility of water in the organic solvent (water/solvent in grams), and *ρ* is the density of *n*-octanol. The emulsion was centrifuged at 10,000 rpm for 2 min to collect the precipitants. Once the supernatant was removed, the samples were filled with ethanol, and we repeated the process twice. To further remove the residual solvents, the precipitant was dried in a freeze dryer equipped with a cold trap set at −80 °C (LP-20; Ilshin Bio Base, Yangju, Korea). The vapor pressure and temperature were set at 25 °C and 50 mTorr or 35 °C and 200 mTorr for 48 h.

### 2.3. Hydrophobic Modification of SPG Membranes and Regeneration

Before hydrophobic modification, the SPG membrane was ignited at 500 °C for 14 h. A muffle furnace (DF-2; Dae Heung Science, Ansan, Korea) was used for ignition. In order to convert the SPG membrane into a hydrophobic one, the SPG membrane was sonicated in a 1.75% solution of an organic silicone resin for 4 h (KP-18C; Shin-Etsu Chemical Co., Tokyo, Japan). The organic silicone resin was diluted with water or ethanol. The converted membrane was dried at 120 °C for 4 h. The process was adopted and modified according to previously reported procedures [26,27,28,29,30,31,32,33]. Details of the different regeneration methods of the SPG membrane are listed in Table 2.

### 2.4. Flow Imaging (FI)

Prepared IVIG microbeads were measured using a Flowcam 8100 (Fluid-imaging Technologies, ME, USA) equipped with a 10 times magnification camera. The camera was calibrated using the 15 µm polystyrene beads provided with the equipment. Before each sample measurement, deionized water was tested for the cleanliness of the fluid path and flow cell (i.e., <100 particles/mL) and then fluxed with ethanol to remove any residual water. For sample preparation, 3 mg IVIG microbeads was dispersed in 1 mL ethanol and then diluted 500 times. In other words, the particles were measured in the form of dried microbeads but re-dispersed in ethanol. For each sample, 1 mL of solution was loaded into the instrument sample port, 0.2 mL was run for priming the system, and then data were achieved for the next 0.2 mL (*n* = 3). The flow rate was fixed at 0.1 mL/min. The analysis was performed according to our previously reported procedures [12,17]. The lowest particle size measured was from 1 μm. Particle sizes and particle concentration were determined as the equivalent spherical diameter (ESD) and the number of particles per mL (p/mL) using the Visual spreadsheet software (version 4.17.14) provided with the equipment, respectively. Three separate measurements were performed for each sample to calculate the mean and standard deviation (SD).

### 2.5. Size-Exclusion Chromatography (SEC)

SEC analysis was utilized using an Agilent HPLC 1260 series (Agilent, Santa Clara, CA, USA) equipped with a diode array detector at an ultraviolet wavelength of 280 nm. The column used was a 30 cm long TSKgel G3000SWXL SEC column (TOSOH Bioscience, King of Prussia, PA, USA) connected with a pre-filter (TridentTM high-pressure in-line filter, Restek, Bellefonte, PA, USA). For the mobile phase, 3× phosphate-buffered saline (PBS, pH 7.4) was used with a flow rate of 0.5 mL/min. The injection volume of the sample was set at 20 µL. Prior to each measurement, the samples were centrifugally filtered. The recovery of IVIG was calculated using the following equation:

Recovery of IVIG % = (*A_s_* ÷ *A*_0_) × 100

where ‘*A_s_*’ is the area of the monomeric peak after rehydration of the microbeads and ‘*A*_0_’ is the area of the monomeric peak of the reference before microbeadification. The analysis was performed according to our previously reported procedure [17].

### 2.6. Scanning Electronic Microscopy (SEM)

The morphology of the IVIG microbeads was observed using an EM-30 SEM (COXEM, Daejeon, Korea) at 20.0 kV acceleration voltage. The microbeads were pre-treated with gold using an SPT-20 ion coater (COXEM, Daejeon, Korea). The analysis was performed according to our previously reported procedure [17].

### 2.7. Statistics

The data are expressed as the mean ± SD. The statistical analysis was utilized using Origin Pro V.2016 software (Originlab Corp., Northampton, MA, USA). Comparisons of means were carried out using a paired *t*-test. A *p*-value < 0.05 was considered as statistically significant.

## 3. Results and Discussion

### 3.1. Effect of the Ejection Time and Repeated Use of the SPG Membrane (Case 1)

**Figure 1:**
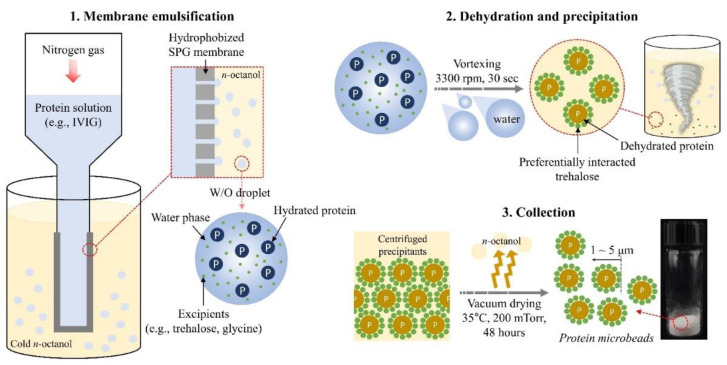
Schematic diagram of protein microbeadification prepared through 1. hydrophobized SPG membrane emulsification technique in cold *n*-octanol followed by 2. dehydration and precipitating using a vortex, and 3. vacuum drying of precipitates after centrifugal collection.

**Figure 2:**
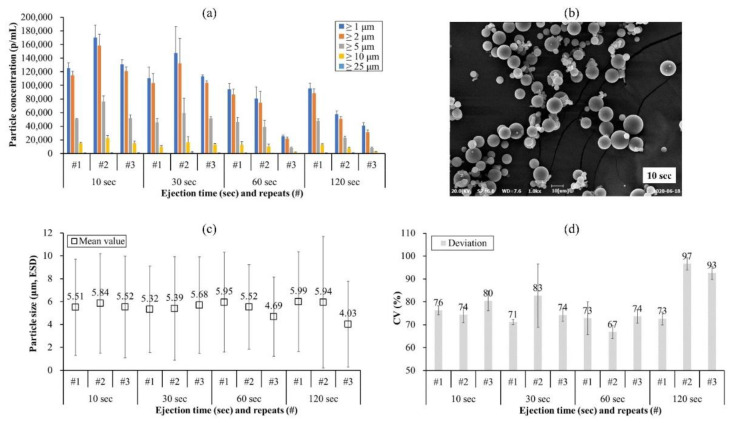
Size distribution of IVIG microbeads prepared by different ejection times and number of repeats expressed in terms of (**a**) particle concentration, (**c**) mean value, and (**d**) CV. The standard deviation of (**a**) and (**d**) was calculated from the average value of three individual measurements, whereas (**c**) was from the total number of particles detected in the FI analysis. (**b**) Microscopic observation of IVIG microbeads prepared with a 10 s ejection time. Regeneration of the SPG membrane was not performed throughout the production.

**Figure 3:**
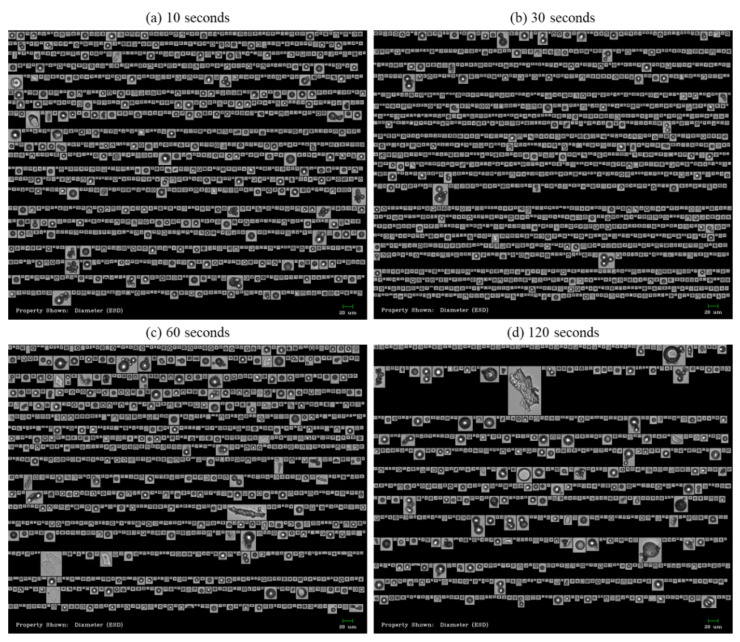
Representative flow images of IVIG microbeads with a diameter greater than 1 μm. (**a**–**d**) exhibit the produced microparticles with different ejection times of 10 s, 30 s, 60 s, and 120 s, respectively. The images obtained were based on equivalent spherical diameter (ESD) and are in the order of detection from left to right and top to bottom.

Figure 1 exhibits the SPG emulsification system to manufacture the IVIG W/O emulsion followed by dehydration and collection to achieve fine IVIG microbeads. The same experimental setup was utilized as in a previous study [17]. In the present study, an additional study was performed to test the process for applicability as a continuous process by (1) extending the nitrogen gas purging (herein termed the ‘ejection time’), forcing the protein solution through the SPG membrane, and (2) repeating the emulsification with the used SPG membrane but with a separate protein solution and *n*-octanol. The volume of the IVIG solution and *n*-octanol was fixed to 0.5 mL and 23.4 mL, respectively. At experimental intervals, only water was used to flush the membrane. After collection of the IVIG microbeads, they were re-dispersed in ethanol to evaluate the particle concentration and size distribution by FI analysis as shown in Figure 2a,c,d and Figure 3.

Overall, the particle concentration (herein size greater than 1 μm) of the IVIG microbeads was affected by both the ejection time and the number of repeats (*n* = 3; expressed as ‘#number’) (Figure 2a). At 10 s, around 100,000 p/mL of IVIG microbeads were detected, but this tentatively decreased when the nitrogen gas was purged longer, by around 2- to 10-fold. Supportively, 5 s and 300 s were also included in the study plan (data not shown) and the particle concentration was the highest at 5 s and lowest at 300 s, reaching around 300,000 p/mL and 16,000 p/mL, respectively. In other words, #1 of 10 s is not the first use of the regenerated SPG membrane but #1 of 5 s. Moreover, the concentrations of #1, #2, and #3 were not consistent at every ejection time, suggesting that re-using the unwashed (i.e., only water flushed) SPG membrane affects the efficiency of microparticle formation, and a longer ejection time might induce a wider size deviation. The SEM image in Figure 2b exhibits a wide size distribution of IVIG microbeads, spherically shaped particles approximately 1 μm to 10 μm. By FI analysis, the mean size of the IVIG microbeads was determined to be from 4 μm to 6 μm (Figure 2c).

The coefficient of variation (CV) was calculated based on the mean value and standard deviation from repeated FI measurements (*n* = 3) to determine the size deviation. This showed an above 70% size deviation for the first three ejection time intervals (i.e., 10 s, 30 s, and 60 s), whereas there was an above 90% size deviation for the last two batches of 120 s (#2 and #3 in Figure 2d). Certain non-spherical and large proteinaceous particles were detected in the FI image at 60 s and 120 s (Figure 3c,d) where a decrease in particle concentration and an increase in size deviation were observed. This could be speculated as being due to the formation of a protein film on the SPG membrane affecting the release of the protein and/or protein aggregation itself causing pore clogging. Due to this fact, the release of protein from the membrane could have differed during every emulsification. Therefore, re-using the SPG membrane was considered not sufficient and extensive work was performed to identify an optimized procedure to regenerate the SPG membrane to enhance its reproducibility.

### 3.2. Effect of Membrane Regeneration on Its Reproducibility (Case 2)

The first case study was repeated after regenerating the SPG membrane with a fixed ejection time of 10 s. Prior to each process, the SPG membrane was regenerated based on the general regeneration method provided with the equipment (wash method A in Table 2). Moreover, the production of IVIG microbeads was repeated daily with and without hydrophobic modification (herein the untreated membrane is termed hydrophilic).

**Figure 4:**
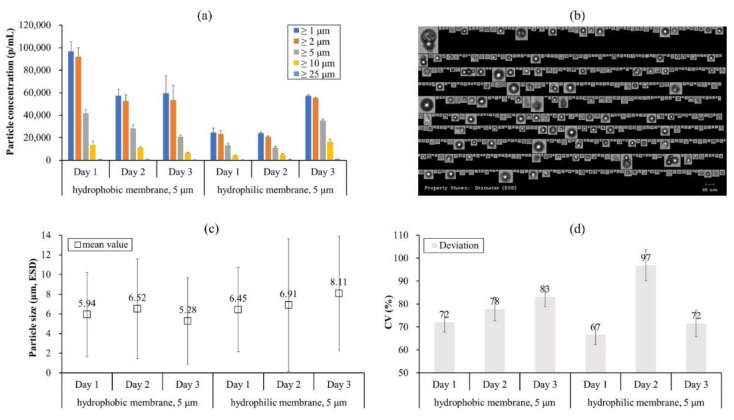
Size distribution of IVIG microbeads prepared by regenerated SPG membrane prior to each production expressed in terms of (**a**) particle concentration, (**c**) mean value, and (**d**) CV. The standard deviation of (**a**,**d**) was calculated from the average value of three individual measurements, whereas (**c**) was from the total number of particles detected in the FI analysis. (**b**) Representative flow image of IVIG microbeads produced by the hydrophilic SPG membrane (i.e., no hydrophobic modification but only washing).

The hydrophobically modified SPG membrane (pore size 5 μm) showed different particle concentrations from day 1 to day 2 and 3, resulting in an inconsistent particle concentration (Figure 4a). Its size deviation increased from 72% to 83% as repeated (Figure 4d). These results suggest that the SPG membrane was not reproducible although it was rinsed and regenerated for each production. Likewise, the hydrophilic SPG membrane also resulted in inconsistent particle concentrations. Its mean sizes were relatively higher, and the highest size deviation was observed on day 2 (Figure 4c,d). This could be due to larger IVIG microbeads (>20 μm) formed during the process (Figure 4b), which could be due to different surface interactions when forming W/O emulsions. Comprehensively, stronger interactions of the W/O emulsion on the hydrophilic membrane caused inconsistent sizes of the droplets. A similar phenomenon was reported earlier, that hydrophobically modified SPG membranes released smaller and monodispersed agarose microspheres (CV = 12.2%) than untreated SPG membranes (CV = 56.3%) [29]. Therefore, the hydrophobic modification of the SPG membrane is necessary and promising for producing fine protein microbeads. Additional studies were performed to solve the inconsistency of the particle concentration and size distribution using the hydrophobically modified SPG membrane.

### 3.3. Modified Wash Method and Pore Size of the SPG Membrane (Case 3)

**Figure 5:**
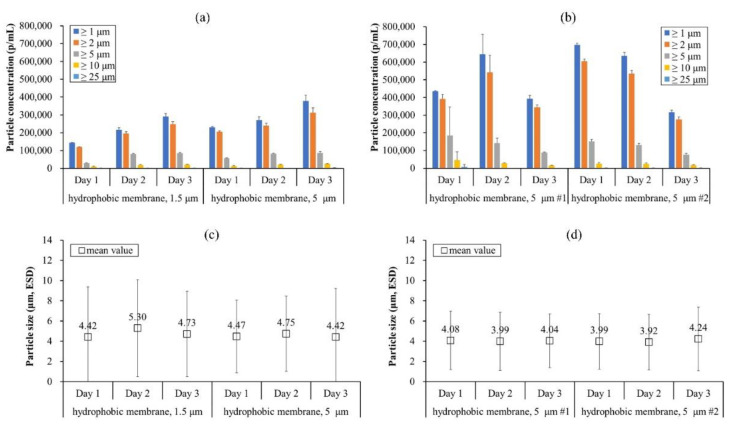
Size distribution of IVIG microbeads expressed in terms of particle concentration and mean value of (**a**,**c**) for case study 3, whereas (**b**,**d**) are for case study 4, respectively. The standard deviation of (**a**,**b**) was calculated from the average value of three individual measurements, whereas (**c**,**d**) are from the total number of particles detected in the FI analysis.

Based on the experience from the first two studies, wash method A was modified by using nitric acid to dissolve any presence of adsorbed proteins on the membrane before hydrophobic modification; this was termed wash method B (Table 2). With the new wash method, IVIG microbeads were reproduced based on the former study as well as with two differently pore-sized SPG membranes (i.e., 1.5 μm and 5 μm). Both membranes had been used to emulsify proteins in the past and were regenerated previously using wash method A. As a result, the particle concentrations were initially over 100,000 p/mL for both membranes and continuously increased to around 200,000–300,000 p/mL as rinsing and regeneration of the membrane were repeated daily (Figure 5a). Considering the lower level of the particles and their decreasing propensity for cases 1 and 2 (Figure 2a and Figure 4a), the case 3 result supports the effectiveness of the new wash method, suggesting that adsorbed proteins on the membrane could have been the cause of its inefficiency.

Overall, the mean size of the IVIG microbeads was decreased to around 4 μm with a narrowed standard deviation (Figure 2c and Figure 4c vs. Figure 5c). However, the pore size of the SPG membrane did not seem to be the size determining factor of the microbeads since the mean size from the 1.5 μm and 5 μm membranes did not differ significantly (*p*-value > 0.05). A lower particle concentration and a wider size distribution were observed using the 1.5 μm pore-sized SPG membrane. This phenomenon could be speculated to be due to the limited flux in the smaller pore size, having a higher flow resistance and colloidal stresses on the proteins, being more adsorptive. Generally, protein adsorption during membrane filtration depends on the pore sizes as well as the membrane materials. An earlier study demonstrated that a 200 nm pore alumina membrane had a greater ability of protein adsorption resistance than a 50 nm pore zirconia membrane but also had more adsorption-related pore plugging [30]. For the SPG membrane, its hydrophobicity did not affect the adsorption properties of BSA except the initial adsorption interaction in the early stage of the filtration [31]. The following study demonstrated the dynamic adsorption behavior of BSA on pre-adsorbed BSA by static adsorption, resulting in multilayer adsorption on the SPG membrane [32]. These studies explain why a proteinaceous film (i.e., non-spherical particles) appeared after repeated usage of the SPG membrane (Figure 3c,d) and support the necessity of a better washing method to remove any adsorbed proteins since simply firing the membrane at 500 °C was not enough, or possibly there was a need to decrease the protein concentration for membrane emulsification.

### 3.4. Two Different Lots and a Decreased Protein Concentration (Case 4 and 5)

The next case study was conducted to confirm the efficiency of the existing 5 μm pore-sized SPG membrane (#1 in Figure 5b) against a brand-new membrane, which was hydrophobically modified, equivalent to wash method A (#2 in Figure 5b). In this case, the loading IVIG concentration was decreased from 100 mg/mL to 50 mg/mL, thereby potentially decreasing the initial adsorption interaction in the early stage of the filtration. In addition, the processing conditions on emulsification (release), dehydration (vortex), and collection (drying) were changed according to the latest optimized process [17] (Table 1). As a result, the particle concentrations were greatly increased to around 300,000–700,000 p/mL (Figure 5b) and its standard deviations of particle sizes were within a narrow range, 1–7 μm (Figure 5d). Nevertheless, the particle concentrations were not consistent from each production, even with the brand-new membrane. It showed the highest particle concentration at the first production, but it decreased when repeated (Figure 5b). This result indicates the insufficiency of wash method B since the particle concentration decreased as the membrane was regenerated.

**Figure 6:**
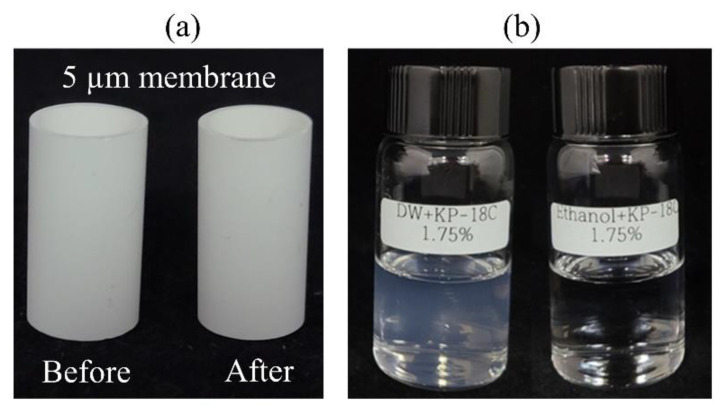
Visual inspection of (**a**) 5 μm pore-sized SPG membrane before and after hydrophobic modification and (**b**) 1.75% silicone resin in deionized water (**left**) and ethanol (**right**).

At this point, the consistency of the hydrophobic modification (Table 1) was doubted; possibly the SPG membrane was not consistently hydrophobized by the silicone resin dispersed in deionized water. As shown in Figure 6a, it was somewhat difficult to judge the completion of the hydrophobic modification, which was based on silicone resin in water (left image in Figure 6b). In addition, the dryness of the membrane was also doubted since the membrane was dried in a furnace without silica gel powder nor relevant accessories to remove any evaporated moisture. Hence, the solvent was substituted with ethanol, and it was dried longer in the furnace (Table 2).

**Figure 7:**
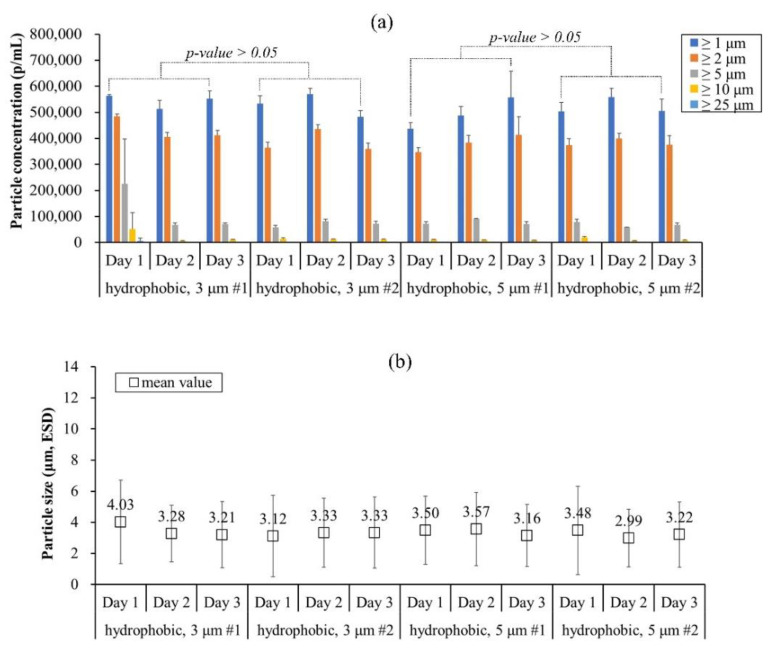
Size distribution of IVIG microbeads prepared by regenerated 3 μm and 5 μm pore-sized SPG membranes in different lots expressed in terms of (**a**) particle concentration and (**b**) mean value. The standard deviation of (**a**) was calculated from the average value of three individual measurements, whereas (**b**) was from the total number of particles detected in FI analysis.

This method was termed wash method C and it was utilized to repeat case 4 using two lots from 3 μm and 5 μm pore-sized SPG membranes. Moreover, the IVIG concentration was diluted to 25 mg/mL (Figure 7). As a result, all particle concentrations were above 400,000 p/mL and the count did not differ significantly as the regeneration was repeated daily on both lots. This means that the SPG membrane emulsification technique must be considered using both a suitable regeneration method and protein concentration. After establishing the reproducibility of the microbeads, a further study was performed to enhance the protein reversibility upon rehydration.

### 3.5. Effect of Cold Ethanol Treatment and Protein Stabilizers (Case 6)

During the collection of the protein microbeads by centrifugation (Figure 1), cold ethanol was used to rinse the precipitants and to reduce the amount of *n*-octanol before the drying process. However, this approach was questioned after the trials and errors in the previous case studies since soluble aggregates or high molecular weight species (HMWs) were observed upon rehydration (see below). This could be due to the incompatibility of ethanol with IgG, similar to losing IgG during ethanol fractionation [33,34]. Perhaps, filtration technology instead of centrifugation for the removal of the continuous phase would be favorable but it was not considered further due to experimental limitations when using a closed system. As a next study, centrifugation for the collection of IVIG precipitants was compared with the presence and absence of a cold ethanol treatment. Prior to membrane emulsification, the IVIG was dialyzed in acetate buffer at pH 4 to remove the glycine and add PS80 and trehalose to observe its stabilizing effect during the use of the ethanol.

**Figure 8:**
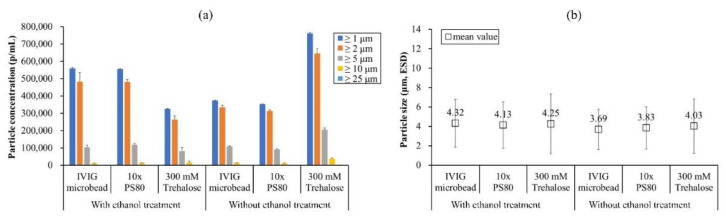
Size distribution of IVIG microbeads prepared with and without cold ethanol treatment expressed in terms of (**a**) particle concentration and (**b**) mean value. The standard deviation of (**a**) was calculated from the average value of three individual measurements, whereas (**b**) was from the total number of particles detected in the FI analysis.

As shown in Figure 8, the particle concentration and the mean size of the IVIG microbeads without the treatment were relatively lower and smaller, respectively. The indicates that the treatment affects the distribution of the microbeads. The difference was the same when PS80 (10 times critical micelle concentration) was pre-added to the IVIG solution, suggesting the difference was not mediated by interfacial stresses. Alternatively, the addition of 300 mM trehalose, already defined as a stabilizer for protein microbead formation and reversibility [17], without the treatment exhibited an increase in the particle concentration of the microbeads up to 700,000 p/mL, whereas with the treatment it decreased down to 300,000 p/mL. These results could be speculated as being due to (1) < 0.05% water content in the ethanol rehydrating some IVIG to aggregate and form insoluble microparticles, and (2) the dissolved trehalose in water or ethanol would lead the microbeads to flocculate, thereby losing counts in the FI measurements.

**Figure 9:**
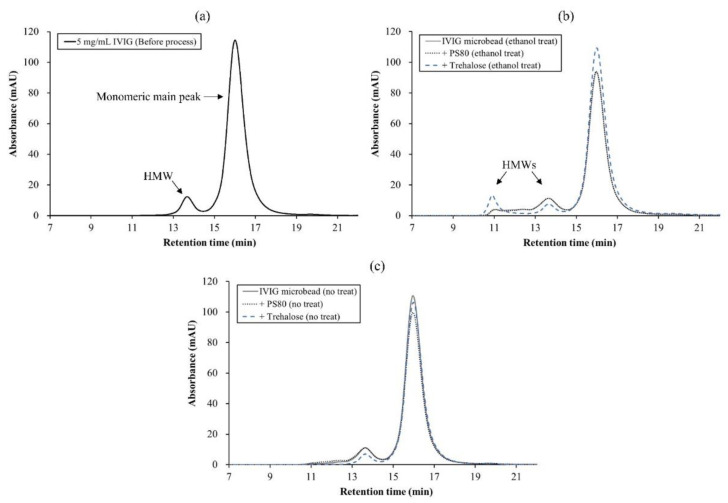
SEC result of 5 mg/mL IVIG (**a**) before microbeadification and after microbeadification (**b**) with and (**c**) without cold ethanol treatment. Deionized water was used to rehydrate the IVIG microbeads.

To evaluate their aggregations and monomeric content, the IVIG microbeads were rehydrated using deionized water and were analyzed by SEC. Figure 9a exhibits a typical size-exclusion chromatogram of 5 mg/mL dialyzed IVIG before microbeadification. It represents two peaks of monomeric and dimeric IgG populations in acetate buffer at pH 4. The shift toward a shorter time represents the formation of larger oligomers or aggregates. After microbeadification of the IVIG with the ethanol, an increased level of HMWs was observed in all three microbeads upon rehydration (Figure 9b). The highest monomer was retained with the addition of trehalose, which also had the highest level of larger HMWs. This could be explained by preferential interactions of trehalose with monomers and oligomers suppressing its unfolding, thereby limiting the formation of insoluble aggregates to a size greater than 100 nm (undetectable by SEC). In previous studies, trehalose increased the folding stability of the native state and reduced the stress-induced aggregation in the bulk solution [35,36,37]. Moreover, it could be attributed to the formation of effective hydrogen bonds with the protein in the absence of water [17,38,39]. Comprehensively, the higher particle concentration of IVIG microbeads without trehalose could be explained as being due to the presence of insoluble aggregates in the micro-size range. The higher particle concentration of the IVIG microbeads with trehalose could be due to higher monomeric IgG being retained during the process. Nevertheless, rinsing the IVIG precipitants with cold ethanol would not be favorable since the lowest level of HMW was observed without the treatment (Figure 9c). The slight difference in the monomeric peak height with and without trehalose (i.e., blue dotted vs. grey solid chromatogram in Figure 9c) was regarded as a minor issue since it could be a gravimetrical error when directly weighing the microbeads before rehydration. For example, the amounts of stabilizers were compensated for in the mass calculation, but its slight loss during the production after weighing might have caused the difference.

### 3.6. Enhanced Reproducibility and Reversibility of IVIG Microbead (Case 7)

**Figure 10:**
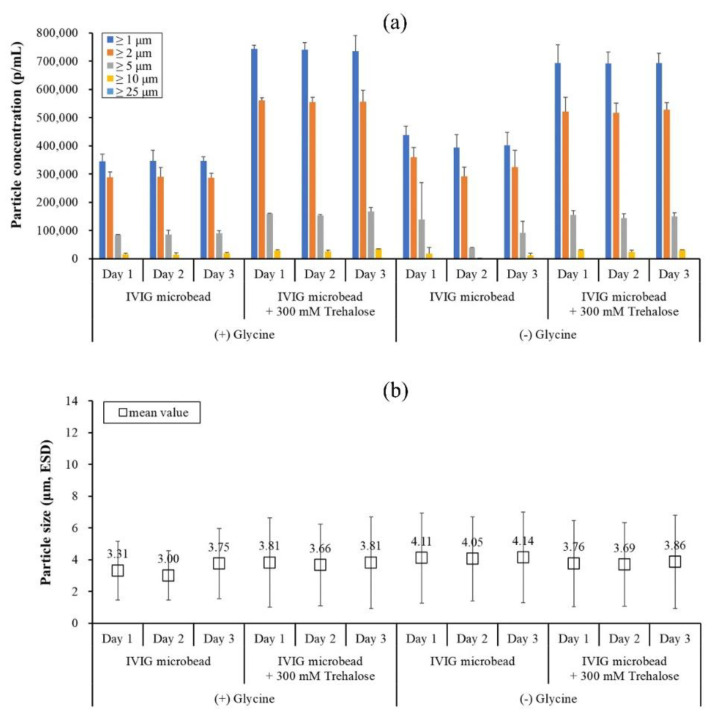
Size distribution of IVIG microbeads prepared with different pharmaceutical excipients expressed in terms of (**a**) particle concentration and (**b**) mean value. The standard deviation of (**a**) was calculated from the average value of three individual measurements, whereas (**b**) was from the total number of particles detected in the FI analysis.

Based on the experiences gained, one last case study was performed to confirm the reproducibility of the IVIG microbeads and its reversibility upon rehydration. To test the reproducibility, different IVIG microbeads were produced using a 5 μm pore-sized SPG membrane regenerated daily by wash method C and without cold ethanol treatment. Four different 25 mg/mL IVIG solutions were tested with the presence and absence of glycine and trehalose. In the case without (−) glycine, the commercial IVIG product was dialyzed in acetate buffer at pH 4, whereas 18.8 mg/mL glycine solution was used as a diluent for the with (+) glycine samples. As shown in Figure 10a, the particle concentration of each of the IVIG microbeads was highly consistent when produced daily, confirming its reproducibility, and enabling precise comparison to one another.

**Figure 11:**
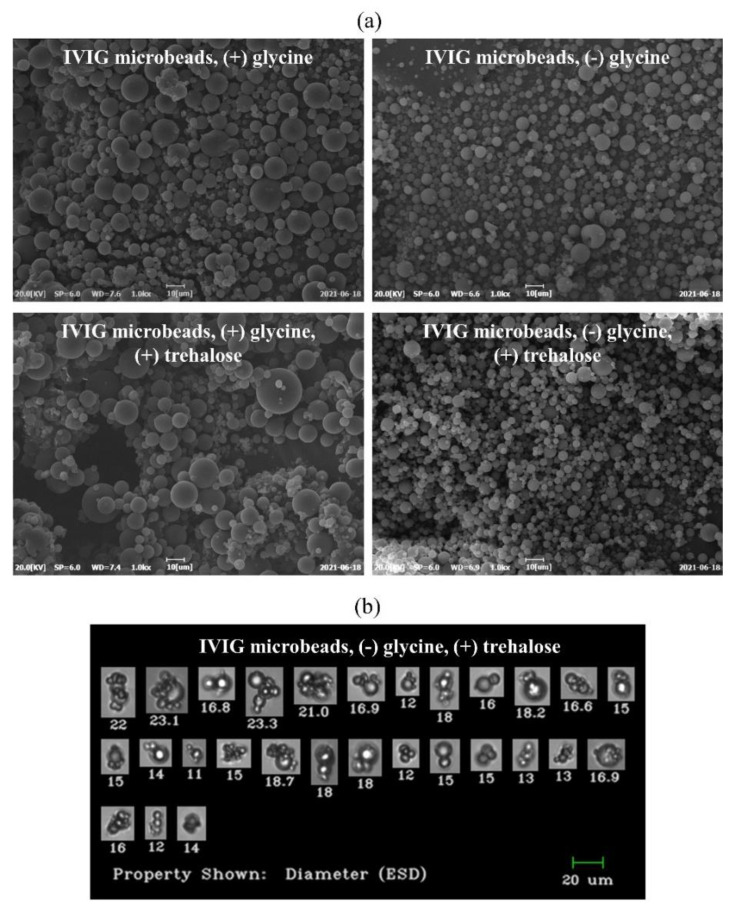
(**a**) Microscopic observation of IVIG microbeads prepared with different pharmaceutical excipients and (**b**) representative flow image of flocculated IVIG microbeads in size greater than 10 μm prepared with trehalose but not glycine.

The particle concentration of the IVIG microbeads with trehalose and glycine was significantly higher by almost 2-fold (*p*-value < 0.05). This result is similar to that in Figure 8a, suggesting trehalose could have suppressed the loss of the monomer into aggregates during the process, and thereby more microbeads were formed. The mean size of each of the IVIG microbeads varied from 3.0 μm to 4.11 μm, and the largest mean size occurred without glycine and trehalose (*p*-value < 0.05). For the size deviation, IVIG microbeads with glycine demonstrated the narrowest distribution on day 1, day 2, and day 3, having a CV of 56%, 52%, and 59%, respectively. With trehalose, its size deviation was increased to 74%, 70%, and 76%, respectively, suggesting a wider distribution due to its cohesive properties. This result could be supported by the clumps of microbeads with the addition of trehalose as shown in the SEM images (Figure 11a), and the clumps or flocculates were detected as single particulates in the FI measurements (Figure 11b). Since the dispersant for the FI measurement was the same ethanol used in the previous rinsing study, the analysis would potentially provide a broader size deviation. In this case, a dry powder size measurement would be more favorable. However, it was not utilized in this study due to the limited amount of microbeads produced for screening (i.e., around 12.5 mg IVIG per production). Nevertheless, the FI measurement was informative about the relative counts as well as for visualization of each particle, distinguishing microbeads, flocculates, and protein film from the SPG membrane (Figure 3c,d). Finally, the prepared IVIG microbeads were rehydrated at a concentration of 100 mg/mL, followed by dilution to 10 mg/mL prior to SEC measurement to evaluate its reversibility after the exposure to high protein concentration.

**Figure 12:**
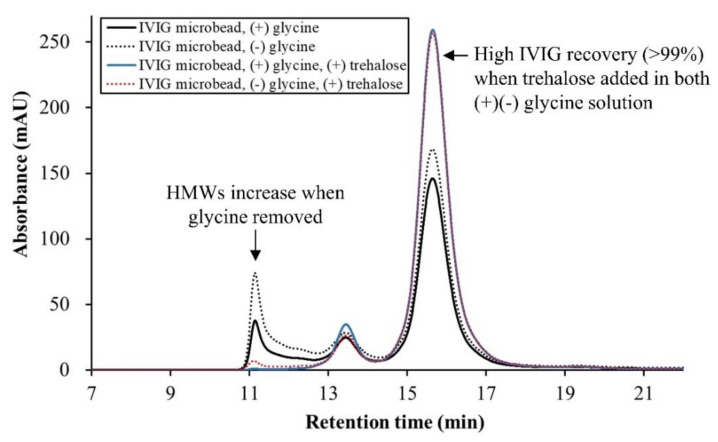
Overlaid SEC result of IVIG microbeads upon rehydration by deionized water. IVIG (100 mg/mL) was diluted 10-fold prior to SEC measurement followed by spin filtration.

At this point, the proteins might meet challenges on rehydration due to their high concentration [40]. As a result, the highest level of monomer was observed for the IVIG microbeads with trehalose, almost double the amount of its monomeric IgG without it. This supports the difference in the particle concentration measured before rehydration. The loss of the monomer was related to the formation of HMWs, which was the highest when neither glycine nor trehalose was added prior to membrane emulsification. The recovery of IVIG microbeads with trehalose was over 99% and seemed more conformationally stable when glycine was included since no sign of a larger HMW was observed. However, relatively smaller HMWs were maintained, expected to be in the dimeric form generally observed with this IVIG product [41]. In summary, the decrease in the monomeric peak of IVIG on SEC chromatogram (Figure 12) represents the denaturation of IVIG during the processing, especially when performed without trehalose and glycine, or even exposed to cold ethanol treatment. As a result, denatured IVIG formed protein aggregates or oligomers upon rehydration. Alternatively, no loss in the monomeric peak of IVIG was observed when the microbead was produced in the optimized process with trehalose and glycine, suggesting the additives suppressed its denaturation during the process.

## 4. Conclusions

In a previous study, protein microbeads were produced using an SPG membrane emulsification technique in *n*-octanol with prompt dehydration [17]. Due to its weighable property as a powder and high reversibility, the microbeads provided a new approach to the protein precipitation process. However, its reproducibility remained unsolved during that study. Generally, membrane emulsification is used to prepare different types of monodispersed particles for small molecules. However, to adopt the technique for biologicals such as proteins or antibodies, their conformational and colloidal stability should never be underestimated since these large molecules are marginally stable and adsorb onto surfaces, especially on SPG membrane during emulsification. Therefore, removing the adsorbed proteins during regeneration of the SPG membrane would be the key to success in its reproducibility and changing the protein concentration to minimize its adsorption. Moreover, the addition of disaccharides into the pre-mix solution would potentially suppress protein unfolding derived from limited water molecules, thereby maximize its reversibility upon rehydration.

## Figures and Tables

**Table 1:** Summary of case studies in the production of IVIG microbeads.

Case	Purpose	Pore Size(μm)	IVIG (mg/mL)	Release (s)	Vortex (s)	Cold Ethanol Treatment	Dry (°C, mTorr)	Regeneration Method ^a^
1	Purge duration	5	100 ^b^	5–300	600	Yes	25, 50	-
2	Repeatability (3-day)	5	100 ^b^	10	600	Yes	25, 50	A
3	Membrane pore size	1.5 and 5	100 ^b^	10	600	Yes	25, 50	B
4	Membrane variation (*n* = 2)	5	50 ^b^	5	30	Yes	35, 200	B
5	Repeatability (3-day)	3 and 5	25 ^b^	5	30	Yes	35, 200	C
6	Trehalose or PS80	5	25 ^c^	5	30	Yes and no	35, 200	C
7	Trehalose and glycine	5	25 ^d^	5	30	No	35, 200	C

^a^: Refer to Table 2; ^b^: with glycine (dilution); ^c^: without glycine (dialysis); ^d^: with and without glycine (dialysis).

**Table 2:** Three different regeneration methods to wash and hydrophobically modify the SPG membrane.

Wash Method	Rinsing	Drying	Hydrophobic Modification
A	Immerse SPG membrane in 50 mL deionized water and sonicate for 20 min (repeat 3 times).	Fired at 500 °C for 14 h in furnace.	Immerse SPG membrane in 20 mL deionized water containing 1.75% silicone resin and sonicate for 4 h. Dried at 110 °C for 2 h.
B	Immerse SPG membrane in 50 mL deionized water and sonicate for 20 min. Immerse SPG membrane in 20 mL nitric acid (60%) and incubate at 70 °C for 20 min (repeat 2 times). Immerse SPG membrane in 50 mL deionized water and sonicate for 10 min (repeat 3 times).		
C	The same as the above wash method 2.		Immerse SPG membrane in 20 mL ethanol containing 1.75% silicone resin and sonicate for 4 h. Dried at 120 °C for 4 h.

## Data Availability

Not applicable.
